# How Do School Salad Bars Impact Elementary School Students’ Dietary Quality and Energy Intake at Lunch? A Randomized Controlled Plate Waste Investigation

**DOI:** 10.3390/nu16234102

**Published:** 2024-11-28

**Authors:** Melanie K. Bean, Suzanne E. Mazzeo, Hollie A. Raynor, Laura M. Thornton, Lilian de Jonge, Ashley Mendoza, Sarah Farthing

**Affiliations:** 1 Department of Pediatrics, School of Medicine, Children’s Hospital of Richmond at Virginia Commonwealth University, Richmond, VA 23298, USA; ashley.cappel@vcuhealth.org (A.M.); sarah.malone@vcuhealth.org (S.F.); 2 Department of Psychology, Virginia Commonwealth University, Richmond, VA 23284, USA; semazzeo@vcu.edu; 3 Department of Nutrition, University of Tennessee, Knoxville, Knoxville, TN 37996, USA; hraynor@utk.edu; 4 Department of Psychiatry, University of North Carolina at Chapel Hill, Chapel Hill, NC 27599, USA; laura_thornton@med.unc.edu; 5 Department of Nutrition and Food Studies, George Mason University, Fairfax, VA 22030, USA; edejonge@gmu.edu

**Keywords:** National School Lunch Program, dietary quality, chronic illness prevention, salad bars, Healthy Eating Index

## Abstract

**Background:** Children’s dietary quality is suboptimal, increasing the risk of numerous chronic illnesses. Salad bars (SBs) have potential to enhance children’s nutritional intake within the National School Lunch Program (NSLP); yet, empirical support is lacking. To address this gap, we evaluated the impact of school salad bars on dietary quality and energy intake at lunch. **Methods:** Seven matched elementary school pairs were randomly selected. All schools served pre-portioned fruit and vegetables (F&V) at baseline. Within each pair, one school received an SB. Digital imagery plate waste methods were applied at baseline and 4–6 weeks post salad bar installation to determine intake (20% increments for food, ounces for beverages). Dietary quality (Healthy Eating Index (HEI-2015)) and energy intake (kcal) were evaluated in NDSR. Multilevel modeling evaluated group (SB vs. control) and time (baseline vs. post) differences and group-by-time interactions for: (1) HEI-2015 (total and component scores) and (2) kcal intake (overall, F&V, non-F&V, and beverage kcals). **Results:** Data from 5674 trays are reported. Significant group-by-time interactions were observed for HEI-2015 total scores and Greens and Beans, Total Fruit, Whole Fruit, Refined Grains, and Added Sugar component scores (*p* < 0.0001), supporting improved dietary quality in SB schools. SB HEI-2015 scores were 60.1 ± 9.8 at post (+5.3 from baseline; *p* < 0.0001) compared with 57.2 ± 9.5 in controls (+1.0 from baseline; *p* = 0.065). Total energy intake significantly increased in SB schools (376 ± 151 kcal (baseline) → 434 ± 176 kcal (post)), driven by F&V energy (+59 kcal), with no change for controls. **Discussion:** Within the NSLP, SBs improved dietary quality and increased energy intake due to increased F&V intake without replacing other foods. Results can inform school nutrition policies designed to reduce chronic illness risk.

## 1. Introduction

Dietary quality is critical to children’s healthy growth and development [1,2]. Yet, the majority of children in the United States (U.S.) consume poor-quality diets, deficient in essential nutrients and high in sodium, added sugar, and saturated fats [3,4]. Indeed, a recent survey indicated that the average overall diet quality of children ages 2–18 was lower than that of all other age groups assessed [5]. Specifically, the mean Healthy Eating Index (HEI) [6] total score (an index comparing diet quality to the current Dietary Guidelines for Americans) for this age group was 54 on a 100-point scale; thus, using HEI interpretation guidelines, U.S. children’s dietary quality has earned a failing grade [7].

The National School Lunch Program (NSLP) is a USD 17.2 billion effort directly targeting children’s dietary quality. Approximately 30.4 million children participate in the NSLP annually [8]. NSLP participation is especially high in schools located in neighborhoods characterized by high levels of poverty; most children in these schools receive free or reduced-price breakfasts and lunches [9,10] and are at particularly high risk for problems such as food insecurity, poor dietary quality, and obesity [11]. Moreover, they consume the majority of their meals within the school setting [11]. Thus, the school food environment is an especially critical setting in which to implement public health efforts addressing dietary quality.

As part of a larger effort to improve school-age children’s dietary quality, the 2010 Healthy Hunger-Free Kids Act (HHFKA) mandated several changes to NSLP meals, including increasing the amounts of fruits and vegetables (F&Vs), whole grains, and low- and non-fat milk offered, while decreasing fats and sodium and establishing age-specific calorie standards [12]. Estimates suggest that the nutritional improvements made with the HHFKA are associated with an estimated 47% reduction in obesity among children living in poverty [13]. Data suggest NSLP participation after HHFKA implementation is linked with better diet quality. Specifically, in 2014–2015, Gearan and colleagues investigated diet quality within a nationally representative sample of Black and white students (*M*_age_ = 12.1; 45.4% elementary students) from lower- and higher-income households [14]. Results indicated that, across income groups, lunches consumed by NSLP participants were higher in dietary quality than those of nonparticipants. NSLP participants’ lunches also included significantly higher concentrations of vegetables, whole grains, and dairy and lower concentrations of sodium, empty calories, and refined grains compared with those of nonparticipants. These results are similar to those of another investigation, which also reported that the dietary quality of lunches consumed by NSLP participants after HHFKA implementation was higher than that of their non-NSLP-participating peers [15]. Overall, these results indicate that the HHFKA is a promising intervention to improve dietary quality; however, a limitation of both of these studies is that consumption was based on self-reported retrospective recall, a method susceptible to both random and systematic error compared with objectively measured consumption [16].

Moreover, although these initial outcomes regarding the HHFKA are promising, additional efforts are needed to enhance children’s dietary quality, as there remains considerable room for improvement. For example, in the study conducted by Kinderknecht and colleagues, cited above, although diet quality (measured by HEI total score at lunch) increased after HHFKA implementation, it was only 54.6 among low-income students at the latter assessment. Scores were similar for low-middle-income and middle-high-income students post-HHFKA (54.8 and 55.5, respectively) [15]. These relatively low scores are concerning, as this and other studies suggest meals eaten outside of school are lower in dietary quality than those consumed within the NSLP [14]. Thus, it is critical to optimize effective strategies to increase children’s dietary quality, including improving the consumption of nutrient-dense foods within school meals.

The installation of salad bars in school cafeterias is one proposed strategy to address this goal. Proponents of school salad bars argue that they are likely to improve students’ dietary quality because they both increase accessibility to a variety of healthy foods and foster students’ ability to make choices about their intake [17]. Although the possibility of installing salad bars in schools is often greeted with enthusiasm, relatively little empirical research has investigated their impact on children’s overall dietary intake [18]. Rather, the few available studies have focused on fruit and vegetable (F&V) intake and not evaluated changes in other nutrients with important health implications, such as whole grains, protein, and added sugar [19,20]. Moreover, the existing investigations have largely been uncontrolled or reliant on self-report measures of intake, which, as noted above, are especially prone to response bias [21,22,23,24].

In addition, more research is needed to examine how salad bars influence energy intake at NSLP lunches. Advocates of school salad bar installation suggest the healthy foods available on the salad bar would displace consumption of calories from less healthy foods [25]. However, empirical data regarding this hypothesis are mixed. One cross-sectional study investigated students’ intake before and after salad bar installation at three elementary schools [23]. Results indicated that F&V intake increased and overall energy consumption decreased, following salad bar installation. However, these results should be interpreted in light of limitations. Specifically, this study had a pre–post design without a control group. Moreover, data were collected in the year 2000, well before HHFKA implementation.

A related evaluation of lunch consumption at nine Wisconsin elementary schools participating in a Farm to School program found that intake from non-FVs decreased as FV consumption increased, providing some evidence of calorie displacement [26]. Total energy intake was highest among students consuming the highest amount of FVs at lunch. However, total average energy intake across all groups was less than the NSLP target (i.e., less than a third of total daily calories). Although these results are promising, this report has limitations, as it was not a controlled study; there was no preintervention evaluation; participants were primarily white and less than half were eligible for free or reduced priced lunch. Moreover, not all schools evaluated had salad bars, and this program was implemented prior to the current NSLP guidelines.

A subsequent investigation assessed the effects of salad bars on FV consumption, after implementation of the HHFKA, in a more diverse setting, using a more rigorous design [27]. Schools in this study had student populations >85% Black or Latinx, and all provided universal free lunches. Schools were matched on demographic characteristics and evaluated in pairs (salad bar/no salad bar). Results indicated that energy intake from vegetables was higher among students in schools with salad bars, compared with peers at non-salad-bar schools. However, no other consistent patterns in energy intake were identified across schools. This investigation improved upon previous uncontrolled evaluations; however, each school was only assessed one time, precluding conclusions about causality.

The current study builds upon and extends prior research in this area by directly examining the impact of salad bar installation on (1) dietary quality of lunches within the NSLP and (2) energy intake overall and from specific meal components, using a controlled, longitudinal design with objective plate waste measures of consumption. Prior evaluations have not examined the influence of salad bars on all the components included in the HEI. Rather, they have focused on F&Vs and have generally not evaluated these components separately. The current investigation capitalizes on a natural experiment, in which schools receiving salad bars for the first time were matched with schools serving standard pre-portioned NSLP meals. It was hypothesized that students at schools with salad bars would consume lunches with higher HEI total scores, as well as higher HEI scores in each of the nine adequacy components, and lower scores on the moderation components. It was further hypothesized that students in salad bar schools would consume less total energy than students in non-salad-bar schools.

## 2. Materials and Methods

### 2.1. Design and Setting

This study was conducted in a sociodemographically diverse Virginia school district, which phased in salad bars to all elementary schools over several years. The investigative team conducted an independent evaluation of this natural experiment, using a wait-list control, cluster randomized controlled design during the 2018–2019 and 2019–2020 school years. Seven elementary school pairs (serving grades K-6), matched on Title I status and % of students from racially and ethnically marginalized backgrounds, were randomly selected. All schools initially served pre-portioned FVs. Within each pair, one school received a salad bar, while the other continued to serve pre-portioned FV. Salad bar menus rotated daily and always included 3 fresh fruits (e.g., sliced pears and halved bananas), 4 vegetables (e.g., salad greens (always included), cucumber slices, tomatoes, seasonal vegetables, and kidney beans), and 2 proteins (e.g., hardboiled egg slices, diced chicken, and shredded cheese). All items were presented separately. Plate waste assessments were conducted at baseline and post (4–6 weeks after the salad bar was installed). Three years of data collection were planned yet were disrupted due to COVID-19. Thus, Years 1 and 2 data for schools that completed baseline and post-test are included. Complete study methods, including characteristics of the included schools, have been previously published [28]. All procedures were approved by the Institutional Review Board of Virginia Commonwealth University.

### 2.2. Participants

Mean enrollment at the participating schools was 632 students (range 367–918) representing diverse backgrounds (35% white, 31% Latinx, 15% Asian American, 11% Black, and 8% more than one race/other race). Six schools were Title I, indicating that they serve low-income families. The average NSLP participation rate was 55% (31–82% across schools), with a mean of 42% (8–75% across schools) of students eligible for free or reduced-price lunch. All students in the selected schools who participated in the NSLP on rating days were eligible. Parents/legal guardians were notified of the study and had the option to opt their child out of plate waste assessments. Children whose parents opted them out of the study were identified with a sticker on rating days. Student verbal assent was also obtained. Fewer than 1% of students opted out (parent or student opt-out). No identifying information was obtained from students (other than grade and gender). All students, regardless of participation, received a small incentive (e.g., fruit-and-vegetable-themed pencil).

### 2.3. Plate Waste Procedures

Validated digital imagery plate waste procedures were conducted, with each school pair rated on the same day for menu consistency at each timepoint. In the school cafeteria, photographs of students’ pre- and post-consumption trays were taken at a 45-degree angle using iPads. Staff placed a numbered label on each tray, indicating grade, gender, and school, and took a “pre-consumption” photograph. After students finished eating, they left their trays on the table. Staff removed napkins and other obstructions, rearranged items so that all were visible in the photograph, poured any remaining beverages into a cup with measurement markings in 0.5 fl oz increments (~15 mL), and took a “post-consumption” image. These images were then uploaded onto computers in the laboratory and the pre- and post-photograph were matched for rating.

### 2.4. Reference Portions

While in the cafeteria, research staff took photographs and direct measurements (using a calibrated food scale, Ozeri Pronto Digital Food Scale (Model ZK14-S)) of three portions of each item served at lunch on rating days. An average weight was used as the reference for a standard portion. Given variable portions of self-serve salad bar items, we used methods described previously to create reference portions of all salad bar menu items [29]. Food was prepared in the laboratory, using methods consistent with those used in the schools (e.g., diced, sliced, or whole). Two dietitians independently served four portions of each item (½c, ¼c, ¾c, and 1c) onto mock cafeteria trays. Portions were weighed in triplicate (after taring to remove the weight of the tray) and an average used as the reference weight. Staff took photographs of these portions for use by raters when estimating starting portions from salad bars.

### 2.5. Laboratory Rating

In the laboratory, trained raters (with inter-rater reliabilities (intraclass correlations [ICC] = 0.94–0.99 at study onset)) determined what foods and beverages were selected, estimated the starting portion of self-serve salad bar items (to the nearest ¼ cup (~59mL)), and recorded the percent left on the plate for all food items (in 20% increments) and fluid ounces remaining for beverages (to the nearest 0.5 fl oz). Salad dressing usage (yes/no) and type (ranch, balsamic vinaigrette, or Italian) was recorded and standard serving sizes (1 oz (~30mL)) applied. Schools used a 24 oz bottle (~710 mL) of each dressing and monitored usage in daily production records. Per the district’s Food and Nutrition Services, 25–35% of students use salad dressing (based on the assumption that 1 oz is used per child), which is consistent with objective assessments in prior research [30]. The average % consumed for all salad bar components was calculated and applied to dressing (if used) to calculate consumption. For other condiments, the number of packets opened and used (to the nearest ½ packet) was indicated (e.g., ketchup). Lunches brought from home were not included. However, foods and drinks from outside of school were rated if (1) the item(s) were supplementing a school-bought lunch; (2) item(s) were present on the tray in both the pre- and post-consumption image; and (3) the image allowed comparable nutrition analyses to be applied (e.g., via observing the brand and/or size of the item). A random sample of ~20% of trays were double rated for quality control.

### 2.6. Nutritional Information

Product information and recipes of all items offered at lunch were obtained from the district dietitian and entered into Nutrition Data Systems for Research (NDSR; version 2019 or 2020, based on year of data collection [31,32]) for analyses. Standard portions (average of three measured portions, as described above) were used. Total nutrition available and consumed were then calculated for each student tray by combining the nutrition data with plate waste data. The amount missing was assumed to have been consumed and subtracted from the plated portion [26,33,34].

The Healthy Eating Index (HEI-2015) scoring algorithm was then applied to each student’s tray to calculate HEI-2015 total and component scores. The HEI-2015 provides a measure of dietary quality compared with current national guidelines based on the Dietary Guidelines for Americans (DGA) [6]. Syntax provided was used to calculate a total HEI-2015 score (possible range = 0–100) and scores for the 13 HEI components. The HEI-2015 contains nine adequacy components (Total Vegetables, Greens and Beans, Total Fruit, Whole Fruit, Whole Grains, Dairy, Total Protein Foods, Seafood and Plant Proteins, and Fatty Acids) and four moderation components (Sodium, Refined Grains, Added Sugars, and Saturated Fat). Higher scores indicate better alignment with the DGA (less intake of moderation components and greater intake of adequacy components).

Energy consumption (kcal) was calculated for the total lunch, fruits and vegetables, non-fruits and vegetables (entrées, snacks, and condiments), and beverages. Whole fruits and vegetables were categorized as fruits or vegetables. Any fruit or vegetable that was part of an entrée (e.g., tomatoes in spaghetti sauce) was captured in entrées and 100% fruit juice was categorized as a beverage.

### 2.7. Statistical Analyses

**Figure 1:**
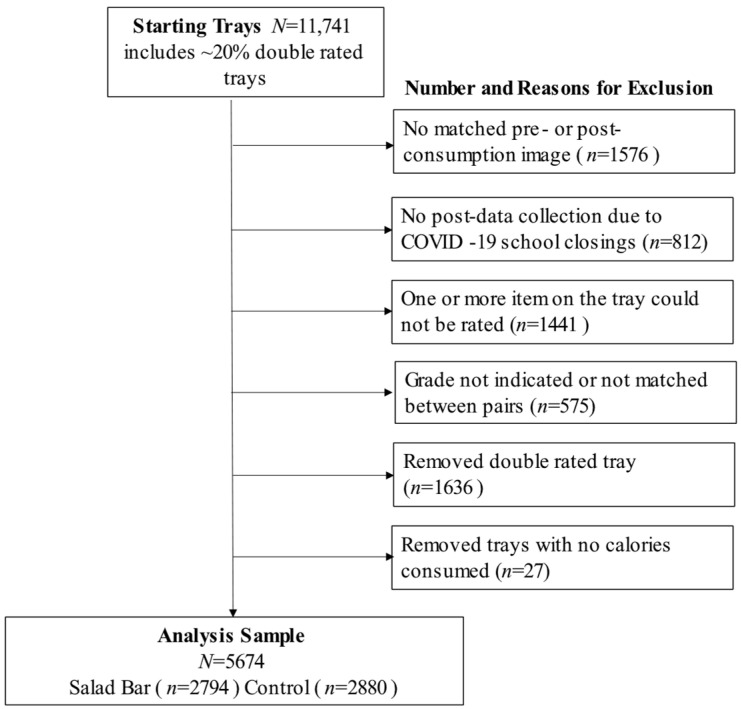
Flow chart of student tray exclusions to yield the final analysis sample. Note: trays that were double rated for quality control were retained until near the final step to maximize the analysis sample; thus, exclusions include double rated trays.

The analysis sample included trays (1) with a matched pre- and post-image, (2) with grade recorded, and (3) for which all meal components could be rated. In addition, trays that had zero energy consumed (*n* = 27) were excluded. Please see Figure 1 for the flow chart of excluded trays to yield the analysis sample (*N* = 5674: salad bar (*n* = 2794); control (*n* = 2880)).

SAS v9.4 was used to conduct all analyses [35]. Descriptive statistics were generated for all variables of interest. Students (trays) are the unit of analysis, which are clustered within schools; thus, these are hierarchical data. Multilevel linear models (MLM) were applied to accommodate the nested data structure by allowing the specification of fixed and random effects and allowing correlated observations within schools. Differences in HEI-2015 total and component scores, total energy, fruit and vegetable energy, non-fruit-and-vegetable energy, and beverage energy between group (salad bar and control), time (baseline and post), and group-by-time interactions were evaluated. All models controlled for school pair and grade (due to differences in energy needs based on age). A Bonferroni correction was applied to account for multiple comparisons (*n* = 18 omnibus tests), setting the *p* for significance at <0.002.

## 3. Results

Please see Table 1 for distribution of student trays by grade and gender. Fewer than 1% of trays (13 salad bar, 29 control) included foods or beverages that supplemented the school meal and typically included fruit, beverages, and snacks. Means (standard deviations) of the HEI-2015 total and component scores and energy intake variables by group (salad bar and control) at baseline (T1) and post (T2) and results of adjusted multivariate models are presented in Table 2.

Mean baseline HEI-2015 total scores were 54.7 ± 10.6 for salad bar schools and 56.6 ± 10.2 for control schools. At post, salad bar students had significantly higher total HEI-2015 scores (60.1 ± 9.8, supporting improvements in dietary quality) compared with controls (57.2 ± 9.5), representing a significant increase (+5.3 points) in salad bar students (*p* < 0.0001). Among the adequacy components, group-by-time interactions were observed for Greens and Beans, Whole Fruit, and Total Fruit, all supporting improved dietary quality in salad bar schools (*p* < 0.0001). Specifically, salad bar schools increased Total Fruit (+1.0 points) and Whole Fruit (+0.8 points), whereas controls had smaller increases in Total Fruit (+0.3 points) and no significant change in Whole Fruit. Significant improvements were observed for Greens and Beans (+0.6 points) in salad bar schools that were not observed in controls. Although there was a significant group-by-time interaction for Total Vegetable and Seafood and Plant Protein scores, there were also significant baseline group differences in these scores, making interpretation difficult. Among the moderation components, significant group-by-time interactions were observed for Sodium, Added Sugar, and Refined Grains, supporting improved dietary quality in salad bar schools (*p* < 0.001). Significant improvements were observed for Added Sugar (+1.0 point) in salad bar schools that were not observed in controls. Both salad bar (+2.4 points) and control (+0.9 points) students improved Sodium scores, with significantly greater increases in the salad bar group.

Total lunch energy intake increased in salad bar schools (+58 kcal), with no change in controls, representing a significant group-by-time interaction (*p* < 0.0001). Evaluation of energy intake by the meal components suggested that the increased energy intake observed in salad bar schools was driven by energy from fruits and vegetables. Specifically, salad bar students consumed an additional 59 kcal from fruits and vegetables at post, with 13 kcal fewer consumed from fruits and vegetables in controls. There were no differences in energy intake from non-fruit-and-vegetable foods or from beverages between groups over time.

## 4. Discussion

This study extended prior work on the effects of salad bars on elementary students’ dietary quality and energy intake. Results indicated that the dietary quality of lunches consumed in schools with salad bars improved compared with that of controls. Specifically, the baseline HEI-2015 total score for lunches consumed in salad bar schools (54.7 on the measure’s 100-point scale) was very close to the national average of 54 found in children ages 2–18 [7]. At post-testing, the HEI-2015 total had increased to 60.1 in salad bar schools. This still represents a “failing” dietary quality grade but is nonetheless a marked improvement. Moreover, the control group’s HEI total did not change significantly over time, supporting the hypothesis that salad bars positively influence dietary quality. In addition, findings suggest that salad bars not only impact F&V intake but also have implications for improved intake of other meal components, as evidenced by HEI-2015 component score improvements in Sodium, Added Sugar, and Refined Grains, in addition to those most directly related to items on the salad bars—Greens and Beans, Whole Fruit, and Total Fruit.

These results have important implications for both children’s short- and long-term health. Although it was traditionally believed that chronic illness risks related to poor dietary quality did not emerge until later adulthood, recent research has indicated that children consuming higher-quality diets have better cardiometabolic health compared with peers with lower-dietary-quality diets [36,37]. Moreover, the increases in dietary quality observed in this trial are likely to have a positive influence on overall health, if maintained. Research with adults has indicated that increasing consumption of fruits, vegetables, and whole grains by a single serving a day is linked to lower all-cause mortality [38]. Moreover, salad bars could play an important role in exposing children to a greater variety of nutrient-dense foods each day than is possible within a pre-set, standard menu. This increased exposure is important, as data suggest children are more likely to consume novel or previously rejected foods after repeated exposures [39,40]. Salad bars can facilitate these repeated exposures while also promoting children’s autonomy to choose their own fruits and vegetables, a factor believed to increase intrinsic motivation for dietary change [39].

Energy intake was higher after installation of salad bars, suggesting that salad bars do not play a direct role in obesity prevention via energy intake reduction. However, calorie reduction within NSLP lunch does not appear to be an appropriate goal, as the consumed lunches already fall below the recommended aim of providing a third of daily expected energy consumption [41]. Indeed, observed energy intake in this study (376–434 kcal) was below the target NSLP calorie requirements for lunch (550–650 kcal) for kindergarten through fifth-grade students [42]. (Of note, these data do not suggest that the district did not serve sufficient calories—yet, due to plate waste, intake was below standards, consistent with previous findings) [43]. Indeed, it seems beneficial that students at salad bar schools were adding energy from nutrient-dense fruits and vegetables and not from other meal components, such as beverages, particularly given that the most common beverage selected is often chocolate milk [44]. This finding is especially important given that prior research has indicated that energy intake within school meals is more nutrient-dense than foods consumed outside of school [14].

As noted, despite improved HEI-2015 scores in salad bar schools, room for improvement remains, with diet quality optimization limited by the outdated meal standards that were in place during this study’s conduct. Specifically, despite the HHFKA’s significant improvements to the NSLP nutrition standards, there have been several subsequent rollbacks and flexibilities (primarily focused on meeting whole grain and sodium targets, permitting flavored milk, and reducing variety of vegetables), weakening dietary quality. Moreover, at the time of this investigation, NSLP standards had not been realigned with the DGAs for over a decade, despite two interim updates to the DGAs to reflect the current evidence base. The Child Nutrition Reauthorization (CNR) provides Congress with an opportunity to strengthen school nutrition standards every five years (consistent with timeline for DGA updates). However, this process has not occurred since the HHFKA, with several failed attempts in Congress, creating missed opportunities to optimize school nutrition and reducing school meals’ potential for disease risk reduction [45]. Additional meal pattern flexibilities were allowed during COVID-19; although essential at the time due to the public health emergency, these changes further compromised nutrition at a time of heightened food and nutrition insecurity [46,47]. Indeed, riders continue to be introduced [48] that would weaken existing standards and roll back proposed improvements, threatening to reverse the progress made by the HHFKA by decreasing the nutritional quality of school meals and compromising the health of millions of children.

Importantly, in 2024, the USDA passed the Final Rule for updating school meal standards (outside of the CNR process) [49]. Changes made include maintaining the whole grain requirement, reinstating the sodium requirement, and, for the first time, including a limit on added sugars. These changes will be phased in starting in the 2025–2026 school year, with full implementation in the 2027–2028 school year. Given these upcoming changes, investigating dietary quality and how salad bars impact dietary quality under these new standards will be important to continue to optimize school nutrition policies and programs designed to improve dietary quality and reduce chronic disease risk.

Current results should be interpreted in light of this study’s limitations. In particular, the assessment of dietary quality and intake conducted in this study focused exclusively on lunch. It was also conducted within a single school district and should be replicated. Nonetheless, this investigation has several strengths, including its large, socioeconomically and racially and ethnically diverse sample, enhancing the generalizability of the observed results. Also, it used objective measures of consumption rated by masked assessors and a well-validated dietary quality measure. Unlike many prior studies in this area, schools were randomly selected, assessed longitudinally, and menus were matched, mitigating the potential influence of confounding factors.

## 5. Conclusions

In sum, these findings suggest that diet quality improved among students in schools with salad bars, with a corresponding increase in total energy intake, due to greater intake of energy from fruits and vegetables. Results support the idea that salad bars can be an important part of a multicomponent strategy to improve the dietary quality of elementary-school-age children. Future research should extend this work to further enhance children’s consumption of nutrient-dense foods, both within and outside of the school setting.

## Figures and Tables

**Table 1:** Sample characteristics of student trays included in evaluation of school salad bars (*N* = 5674: Salad Bar (*n* = 2794) and Control (*n* = 2880)).

		2018–2019 School Year		2019–2020 School Year	
		Salad Bar *n* (%)	Control *n* (%)	Salad Bar *n* (%)	Control *n* (%)
**Baseline**					
Grade	K	81 (15.7)	163 (24.8)	120 (18.8)	155 (22.5)
	1	100 (19.4)	130 (19.8)	118 (18.5)	146 (21.2)
	2	94 (18.2)	132 (20.1)	105 (16.5)	140 (20.3)
	3	85 (16.6)	114 (17.3)	77 (12.1)	108 (15.6)
	4	69 (13.4)	47 (7.1)	94 (14.7)	54 (7.8)
	5	70 (13.6)	61 (9.3)	84 (13.2)	69 (10.0)
	6	16 (3.1)	11 (1.7)	40 (6.3)	18 (2.6)
Gender ^a^	Boy	263 (51.1)	313 (47.6)	307 (48.1)	321 (46.5)
	Girl	252 (28.9)	345 (52.4)	331 (51.9)	369 (53.5)
**Post**					
Grade	K	162 (20.0)	106 (14.7)	161 (19.4)	133 (16.4)
	1	160 (19.7)	95 (13.2)	165 (19.9)	117 (14.4)
	2	164 (20.2)	121 (16.8)	150 (18.1)	118 (14.5)
	3	132 (16.3)	150 (20.9)	141 (17.0)	158 (19.4)
	4	84 (10.3)	97 (13.5)	90 (10.9)	108 (13.3)
	5	72 (8.9)	91 (12.7)	81 (9.8)	97 (11.9)
	6	38 (4.7)	59 (8.2)	41 (5.0)	82 (10.1)
Gender ^a^	Boy	419 (51.6)	363 (50.5)	391 (47.2)	418 (51.4)
	Girl	393 (48.4)	356 (49.5)	438 (52.8)	395 (48.6)

^a^ Gender was determined by raters based on observations.

**Table 2:** Healthy Eating Index-2015 (HEI-2015) total and component scores and energy intake from lunch at baseline (T1) and post (T2) in salad bar and control schools (*N* = 5624).

	Salad Bar (SB)			Control (C)			T1 *p*-Values SB v C	T2 *p*-Values SB v C	Group-by-Time F (*p*-Value)
HEI-2015 Score (Max)	Baseline (T1) *N* = 1327 M (SD)	Post (T2) *N* = 1467 M (SD)	T1–T2 *p*-Value	Baseline (T1) *N* = 1377 M (SD)	Post (T2) *N* = 1503 M (SD)	T1–T2 *p*-Value			
HEI Total Score (100)	54.73 (10.62)	60.07 (9.83)	**<0.0001**	56.26 (10.2)	57.24 (9.52)	0.065	0.091	**<0.0001**	**65.26 (<0.0001)**
Total Vegetables (5)	1.50 (1.85)	1.82 (2.09)	0.006	2.04 (2.03)	1.77 (1.95)	0.002	**<0.0001**	0.768	**28.23 (<0.0001)**
Greens and Beans (5)	0.36 (1.28)	1.00 (1.93)	**<0.0001**	0.30 (1.17)	0.29 (1.15)	1.00	0.667	**<0.0001**	**65.57 (<0.0001)**
Total Fruit (5)	2.65 (2.20)	3.66 (2.05)	**<0.0001**	2.59 (2.14)	2.90 (2.12)	**0.001**	0.994	**<0.0001**	**40.35 (<0.0001)**
Whole Fruit (5)	3.01 (2.33)	3.81 (2.08)	**<0.0001**	3.08 (2.28)	2.97 (2.29)	0.816	0.813	**<0.0001**	**56.30 (<0.0001)**
Whole Grains (10)	8.71 (2.97)	8.30 (3.15)	0.002	8.92 (2.82)	8.38 (3.28)	**<0.0001**	0.461	0.936	0.52 (0.470)
Dairy (10)	8.26 (3.45)	7.75 (3.77)	**0.001**	8.20 (3.54)	7.47 (4.06)	**<0.0001**	1.00	0.914	0.22 (0.637)
Total Protein Foods (5)	2.66 (2.33)	3.10 (2.05)	**<0.0001**	2.51 (2.36)	3.13 (2.16)	**<0.0001**	0.072	0.653	1.10 (0.296)
Seafood and Plant Proteins (5)	1.00 (1.94)	0.57 (1.54)	**<0.0001**	0.70 (1.69)	0.91 (1.87)	0.035	**<0.0001**	**0.001**	**43.99 (<0.0001)**
Fatty Acids (10)	4.33 (4.37)	4.22 (4.16)	0.981	5.41 (4.32)	5.77 (4.27)	0.030	**0.001**	**<0.0001**	3.25 (0.072)
Sodium (10)	5.52 (3.47)	7.92 (2.95)	**<0.0001**	5.72 (3.44)	6.64 (2.86)	**<0.0001**	0.160	**<0.0001**	**72.82 (<0.0001)**
Refined Grains (10)	8.63 (3.03)	9.02 (2.73)	0.013	8.83 (2.91)	8.63 (3.21)	0.365	1.00	**<0.0001**	**13.03 (0.001)**
Added Sugars (10)	7.45 (2.80)	8.35 (2.26)	**<0.0001**	7.46 (2.88)	7.76 (2.53)	0.177	0.719	**<0.0001**	**21.53 (<0.0001)**
Saturated Fats (10)	0.66 (2.31)	0.57 (2.15)	0.748	0.51 (2.08)	0.59 (2.16)	0.798	0.275	0.999	2.24 (0.135)
**Energy Intake**									
Total Energy, kcal	376 (151)	434 (176)	**<0.0001**	385 (152)	386 (158)	1.00	0.277	**<0.0001**	**51.55 (<0.0001)**
FV Energy, kcal	41 (42)	100 (79)	**<0.0001**	59 (53)	46 (44)	**<0.0001**	**<0.0001**	**<0.0001**	**597.33 (<0.0001)**
Non-FV Energy, kcal	269 (135)	272 (150)	0.961	262 (131)	275 (142)	0.112	0.542	0.985	1.49 (0.222)
Beverage Energy, kcal	66 (44)	61 (46)	0.079	64 (45)	65 (47)	0.958	0.906	0.113	4.25 (0.040)

Note: higher HEI scores indicate better alignment with Dietary Guidelines for Americans (greater intake of adequacy components and lower intake of moderation components). Results of multilevel models, adjusted for grade and school pair, shown. Group and time F (*p*-values) results available in Supplementary Table S1. *p* < 0.002 indicates statistical significance, with significant results bolded.

## Data Availability

Data are not yet available as study analyses are still ongoing. The datasets used in the current study will be available from the corresponding author on reasonable request.

## Supplementary Materials

The following supporting information can be downloaded at: https://www.mdpi.com/article/10.3390/nu16234102/s1, Table S1: Results from multilevel models evaluating differences in dietary quality and energy intake between salad bar and control schools (*N* = 5674).
