# Supplementary material for: How Do School Salad Bars Impact Elementary School Students’ Dietary Quality and Energy Intake at Lunch? A Randomized Controlled Plate Waste Investigation
Source: Nutrients. 2024 Nov 28;16(23):4102. doi: 10.3390/nu16234102 (PMC11643552; doi:10.3390/nu16234102)
Supplement: Supplementary file 1 [file nutrients-16-04102-s001.zip › nutrients-3331353-supplementary.pdf]

Table S1. Results from multilevel models evaluating differences in dietary quality and energy intake between salad bar and control schools (N=5,674)

| **HEI-2015 Scores** | **Group F (*p*-value)** | **Time F (*p*-value)** |
|---|---|---|
| HEI-2015 Total Score | **18.07 (<.0001)** | **140.27 (<.0001)** |
| Total Vegetables | **13.93 (.001)** | 0.07 (.790) |
| Greens and Beans | **94.13 (<.0001)** | **60.14 (<.0001)** |
| Total Fruit | **43.73 (<.0001)** | **159.22 (<.0001)** |
| Whole Fruit | **35.20 (<.0001)** | **37.63 (<.0001)** |
| Whole Grains | 2.54 (.112) | **40.20 (<.0001)** |
| Dairy | 0.28 (.578) | **45.11 (<.0001)** |
| Total Protein Foods | 7.65 (.006) | **104.93 (<.0001)** |
| Seafood and Plant Proteins | 0.24 (.625) | 6.25 (.013) |
| Fatty Acids | **61.41 (<.0001)** | 5.80 (.016) |
| Sodium | **25.80 (<.0001)** | **391.16 (<.0001)** |
| Refined Grains | 9.99 (.002) | 1.22 (.269) |
| Added Sugars | 8.26 (.005) | **60.04 (<.0001)** |
| Saturated Fats | 1.85 (.175) | 0.01 (.935) |
| **Energy Intake** | | |
| Total Energy (kcal) | **19.77 (<.0001)** | **49.71 (<.0001)** |
| FV Energy (kcal) | **130.43 (.0001)** | **254.54 (<.0001)** |
| Non-FV Energy (kcal) | 0.51 (.476) | 3.71 (.055) |
| Beverage Energy (kcal) | 1.09 (.296) | 1.82 (.178) |

Note: HEI=Healthy Eating Index-2015; FV=Fruits and Vegetables; models were adjusted for grade and pair; *p*<.002 (bolded) indicates statistical significance
